# Literary Notes

**Published:** 1896-02

**Authors:** 


### Literary Notes.

When novelists undertake medical subjects they should be very well coached and the final proof should be read by a doctor. In "Katharine Lauderdale," Marion Crawford makes one of his subjects die of opium poisoning, with widely dilated pupils. It may be that a man can die that way after having taken a fatal dose of opium, but there is no medical authority for the opinion.—*Post-Graduate.*

---

THE *Medical Fortnightly*, of St. Louis, has issued a handsomely illustrated brochure entitled, The happy medium. The book contains thirty-two pages, incased in a unique embossed cover, and twenty-five half-tone portraits of its staff, including Dr. Frank Parsons Norbury, managing editor; Drs. Hubert Work and T. A. Hopkins, associates; Charles Wood Fassett, secretary, and twenty-one department editors. It is dedicated to friends, patrons and subscribers who are interested in the personnel of the *Fortnightly* staff. It is an agreeable souvenir of an enterprising medical journal.

---

THE *Cleveland Journal of Medicine* is the name of a new medical journal established in Cleveland by the editors of the *Western Reserve Medical Journal*, which latter retires from the field to give place to this its successor. The new journal is edited and published by Henry S. Upson, M. D., and P. Maxwell Foshay, M. D., the latter being business manager. It is the official organ of the Cleveland Medical Society, and the following-named are associate editors on the part of the society: Wm. H. Humiston, M. D., chairman, 122 Euclid avenue; Wm. F. Brokaw, M. D., secretary of society; W. H. Buechner, M. D.; M. Rosenwasser, M. D.; R. M. Woodward, M. D., M. H. S.; William E. Wirt, M. D., president of society. We congratulate all concerned in the new journal and bespeak for it that success which the importance of Cleveland as a medical center and the high standing of its physicians merit.

---

THE history of anesthesia and painless surgery is the title of an interesting little brochure which is a reprint of four papers written by Dr. William R. Hayden, of Bedford Springs, Mass., and published in the *International Journal of Surgery*. Dr. Hayden who is a member of the present Massachusetts legislature, is a firm believer in the claims of the friends of Dr. Morton as to the priority of the latter in the discovery of the anesthetic principle. He sets forth his convictions in a clear and convincing manner. It is more than probable that the Massachusetts legislature, during its present session, will take appropriate action toward doing honor to the memory of Dr. Morton. It is stated that the Massachusetts

General Hospital will make a grand demonstration on October 16, 1896, the semi-centennial of the discovery of anesthesia, in which the hospital authorities will be aided by the great body of the medical and dental professions of the state. The price of this little book is 25 cents and it may be obtained by addressing the author.

---

THE Medical Department of the University of Buffalo has issued a semi-centennial catalogue of its alumni. It is a handsome brochure of forty-four standard octavo pages, printed on book paper, and is a credit to its compiler, who, we presume, is Dr. John Parmenter, though there is nothing in the book to indicate who is its author. Very few colleges are able to present such a catalogue, and it will be prized by the alumni as a valuable record.

---

OUR good friend, the editor of the *Medical Mirror*, in the issue of his estimable journal for December, 1895, has placed us under renewed obligations by his kindly words and wishes, for which we beg him to accept our best thanks. Here is what Dr. Love says:

> We congratulate Dr. William Warren Potter and his associate editors upon the new dress of the BUFFALO MEDICAL JOURNAL. Good taste, solidity and worth appears throughout the pages. This journal, unlike many others, is not satisfied with the fact that it was founded by one of the masters of the medical profession of America and with a creditable record of more than fifty years, but makes constant effort to keep in the front rank. The national prominence of Dr. Potter as president of the American Association of State Board Medical Examiners and as one of the wheel-horses of the medical profession in New York state, cannot fail to be of immense value to the BUFFALO MEDICAL JOURNAL.
>
> We should be glad to see every doctor in America a subscriber and every advertiser represented in the advertising columns.

---

*Popular Science*, a journal of news, invention, botany, chemistry, medicine and the like, appeared in an enlarged and improved form in January, 1896, which was its thirtieth anniversary issue. New departments on invention and electricity have been added, each under the direction of an expert. The number of pages also have been doubled. We congratulate this excellent journal on its handsome and prosperous appearance.

---

THE *Journal of Experimental Medicine* was announced to appear in January, 1896. It is stated that at least four numbers will be published during the year ; at all events, whenever sufficient material, is ready a number of the journal will be issued. It will be devoted to original investigations in physiology, pathology, bacteriology, pharmacology, physiological chemistry, hygiene and medicine. Dr. William H. Welch, 935 St. Paul street, Baltimore, professor of pathology in the Johns Hopkins University, is to be the editor of the new journal and with him will coöperate a board of twelve associate editors, with whom a large number of medical gentlemen have consented to assist as collaborators. The subscription price will be $5.00 a volume. D. Appleton & Co., New York, are its publishers.

---

THE *Medical News* is now published in New York instead of Philadelphia. Nothing now could surprise us unless the *American Journal of Medical Sciences* should run over to Chicago and take up its abode. We have been accustomed for so many years to associate the *News* with Philadelphia that it seems awkward to readjust our mental geography and place it in New York. It will seem odd, too, to separate Dr. Gould's name from the editorial tripod of the *News* ; but we suppose we may as well accustom ourselves to the change first as last, so in bidding good-bye to Dr. Gould, we extend a cordial welcome to Dr. Goffe.

---

THE *Times and Register*, not to be outdone by its migratory Philadelphia contemporaries, hies itself off to Boston, where it is to be edited, though it will still be published, or rather printed, in Philadelphia. It henceforth will appear bi-weekly—twenty-six times a year.

